# Risk Factors Associated with Weight Gain during Treatment with Dupilumab among Patients with Moderate to Severe Atopic Dermatitis

**DOI:** 10.2340/actadv.v104.40796

**Published:** 2024-11-15

**Authors:** Mahsa TAYEFI, Axel SVEDBOM, Lina U. IVERT, Maria LUNDQVIST, Jorge L. RUAS, Maria BRADLEY, Emma K. JOHANSSON

**Affiliations:** 1 Division of Dermatology and Venereology, Department of Medicine Solna, Karolinska Institutet, Stockholm, Sweden; 2 Department of Dermatology and Venereology, Karolinska University Hospital, Stockholm, Sweden; 3 Molecular and Cellular Exercise Physiology, Department of Physiology and Pharmacology, Biomedicum, Karolinska Institutet, Stockholm, Sweden; 4 Department of Pharmacology and Stanley and Judith Frankel Institute for Heart & Brain Health, University of Michigan Medical School, Ann Arbor, MI, USA

**Keywords:** atopic eczema, biologics, side effects, weight gain, body mass

## Abstract

This cohort study used prospectively collected data from the Swedish national quality registry, SwedAD, to investigate weight gain as a possible side effect of dupilumab treatment for atopic dermatitis. Patients on dupilumab were compared with patients on other systemic medications, e.g., methotrexate, cyclosporine, or Janus kinase inhibitors, and possible risk factors for weight change during treatment with dupilumab were analysed. All patients aged 18 years or above, included in SwedAD between March 2018 and April 2023, who initiated systemic treatment at or after inclusion and had data on weight at baseline and at least 1 follow-up weight measurement were included (*n* = 157). After 2 years on dupilumab, patients had a mean weight gain of 1.6 kg (*p* = 0.007, 95% confidence interval [CI] 0.4–2.7). In the multivariable analysis, controlling for age at start, sex, asthma, and body mass index at start, dupilumab was associated with higher weight gain than other systemic treatments (3.3 kg, *p* = 0.005 [95% CI 1.0–5.6]). Asthma was associated with weight loss; male sex tended to be associated with weight gain.

## SIGNIFICANCE

Atopic dermatitis is the most common chronic inflammatory skin disease. Dupilumab, a biological treatment, became available in 2017. Using data from the Swedish national quality registry, SwedAD, we found that dupilumab treatment for atopic dermatitis was associated with weight gain. We found a 3.3 kg weight gain among patients on dupilumab compared with patients on other systemic treatments after 2 years. Patients with self-reported asthma lost weight. Male patients tended to gain more weight than female patients.

Atopic dermatitis (AD) is the most common chronic inflammatory skin disease, with a prevalence of approximately 20% in children and 10% in adults (1, 2). AD presents as dry, red, itchy, and sometimes oozing patches in the skin. Its severity and duration may vary during life and patients with moderate to severe AD experience a negative impact on quality of life, including psychiatric comorbidities (3, 4). Fortunately, in the last decade, there has been a rapid development of new and effective treatments for AD. In Sweden, 2 interleukin (IL) inhibitors, dupilumab and tralokinumab, and 3 Janus kinase (JAK) inhibitors, abrocitinib, baricitinib, and upadacitinib, have been approved for moderate to severe AD. Dupilumab, a human monoclonal antibody against the IL-4α receptor, was the first of the biologics, approved in Sweden in 2017. It reduces T-cell-driven inflammation, specifically T-helper cells (Th2), by inhibiting the IL-4α receptor, and thereby IL-4 and IL-13 signalling (5, 6). It is often the preferred choice in AD patients with other atopic comorbidities, such as asthma (7). JAK inhibitors are targeted immunomodulators that reduce the inflammatory response by binding intracellularly to JAKs and signal transducer and activator of transcription proteins, which are signal transducers for cytokine receptors on keratinocytes and other cells (8). Baricitinib was approved in Sweden in 2020, with upadacitinib and abrocitinib approved soon thereafter.

Real-world data can be key in an exploration of how patients react to treatment in terms of tolerance and efficacy. By using the Swedish nationwide AD registry (SwedAD) to capture patients treated with systemic therapies, we can discover unexpected side effects in daily clinical practice. Earlier findings from SwedAD data showed weight gain in a small cohort of AD patients after 1 year of treatment with dupilumab (9). The mechanism behind the weight gain during treatment with dupilumab is not fully understood. Although researchers suspect that a reduction of inflammatory cytokines causes an increase or redistribution of fat mass and gain in lean mass, more research is needed (10, 11). One possible mechanism by which dupilumab could cause weight gain in AD patients would be by interfering with adipose tissue metabolism. Mouse studies have shown that a reduction in brown adipose tissue is associated with obesity. Upregulation of IL-4 and IL-13 in mouse studies increased active brown adipose tissue, whereas depletion of IL-4 and IL-13 reduced brown adipose tissue. Preventing the action of IL-13 was correlated with more epididymal, inguinal, and perirenal white adipose tissue in mice, but no excessive consumption of food (12). Dupilumab also reduces the activation of IL-4 and could thereby decrease the levels of indole compounds and kynurenic acid (KA) (13), which have been shown to increase adipose tissue energy expenditure and reduce circulating triglyceride levels (14).

This study aimed to investigate weight change in AD patients undergoing dupilumab treatment and explore possible associated risk factors. The primary outcome was mean weight change after 2 years. Additionally, we compared weight change between patients receiving dupilumab with patients on other systemic treatments (e.g., methotrexate, cyclosporine, JAK inhibitors). To explore possible mechanisms behind the weight change, we analysed the concentrations of tryptophan/kynurenine metabolites (including KA) in serum before and during treatment with dupilumab.

## MATERIALS AND METHODS

### Study design

In this cohort study, we used prospectively collected data from the research registry for AD at Karolinska University Hospital, which has been active since 2017. It was converted into a Swedish national quality registry, SwedAD, in 2019 (15). All dermatological units in Sweden have access and can include patients. Both adults and children with moderate to severe AD and eligible for systemic treatments, e.g., methotrexate, cyclosporine, IL inhibitors, or JAK inhibitors, can be included in the registry. SwedAD is used in most dermatological clinics in daily practice, as this is encouraged by the Swedish healthcare authorities. To date, it includes approximately 1,700 patients.

Ethical approval was received from the Regional Ethical Review Board at Karolinska Institutet, Stockholm (2010-345-32 2, 2022-06917-02, 2022-01853-01). Informed written consent was provided by all participants in the research registry.

At the start of any systemic treatment, baseline data are collected. Outcome measurements are performed throughout the treatment period. At baseline, data on body mass index (BMI), age, sex, comorbidities (diabetes, hypertension, asthma, allergy, stroke, myocardial infarction), education, alcohol use, smoking, current location of the eczema, and treatments are collected. Eczema Area and Severity Index (EASI) (16) score is assessed by the treating physician and entered in the registry. In addition, patient-reported outcome measures (PROMs) are collected, e.g., Patient-Oriented Eczema Measure (POEM) (17), Montgomery-Åsberg Depression Rating Scale-Self-report (MADRS-S) (18), Dermatology Life Quality Index (DLQI) (19), and visual analogue scale (VAS) scores were registered at each visit before September 2019, with VAS replaced by the pruritus numeric rating scale (NRS) after September 2019 (20). Patient data were collected at Karolinska University Hospital at the start of treatment, and subsequently at 1 month, 3 months, and every third to sixth month throughout the treatment. However, it is important to note that follow-up intervals may differ across clinics in Sweden, and that baseline data are not mandatory, resulting in potential variations in data coverage.

A small pilot study investigating the association between weight gain and changes in tryptophan/kynurenine metabolites in patients with dupilumab was performed. If a patient had provided a blood sample before starting treatment and was still on treatment, they were asked to give a second sample for the analysis of trypto-phan/kynurenine metabolites in September–October 2022. The included patients (*n* = 13) had been on treatment with dupilumab for 16–60 months.

### Inclusion and exclusion criteria

All AD patients 18 years or older included in SwedAD between March 2018 and April 2023, who initiated systemic treatment at or after inclusion and had data on weight at baseline and at least 1 follow-up weight measurement were included in the cohort. Patients with follow-up < 3 months were excluded. Patients treated with dupilumab were compared with those treated with other systemic drugs (Fig. S1).

### Definitions

All data at baseline, except weight, height, and EASI, were self-reported. This includes data on asthma, allergy, education, heredity for AD, and other atopic manifestations in close family members, and POEM, DLQI, VAS/NRS, and MADRS-S scores.

Weight was measured in kilograms (kg) at the start of treatment and at follow-ups (with intervals depending on the routines at the recruiting clinic).

Education level was categorized into three groups: ≤ 9, 9–12, and ≥ 12 years.

To estimate appetite, we used question 4 in MADRS-S: “Here, you should indicate how your appetite has been and try to recall whether it has differed in any way from normal. If your appetite has been greater than usual, you should mark the scale at zero (0). Scale 0–6; 0: Normal or increased appetite, 2: Slightly reduced appetite, 4: No appetite/food is tasteless, 6: Need persuasion to eat.”

Disturbed night sleep due to itch was defined based on responses to the question “How many nights’ sleep in the last week have been disturbed due to eczema? Scale 0–6; 0: No nights, 1: 1–2 nights, 2: 3–4 nights, 3: 5–6 nights 4: Every night.”

Intensity of pruritus was self-reported on a scale from 0 to 10, measured with VAS or NRS.

### Blood analyses

Plasma concentrations of kynurenine pathway metabolites were measured by Bevital AS (http://www.bevital.no/); a detailed description of the analytical method used has been published elsewhere (21). Sixty µl of plasma was mixed with an equal volume of trichloroacetic acid (60 g/l in water) and centrifuged. The resulting supernatant was injected onto a reverse phase high-performance liquid chromatography column with a gradient mobile phase consisting of water, acetic acid, acetonitrile, and heptafluoroacetic acid. An API4000 tandem mass spectrometer from Sciex (https://sciex.com/) was used for detection of KA and isotope-labelled KA (serving as an internal standard) using electrospray ionization in the positive mode.

### Statistical analysis

The baseline characteristics of the patients are presented as percentages of the total in the group or mean or median values with interquartile ranges. Weight and BMI are shown as mean values with standard deviations (SDs).

The weight change in patients on dupilumab was calculated using linear regression with bivariable analysis and multiple imputations for the variables BMI, age at start, VAS/NRS, EASI, POEM, DLQI, and MADRS-S. Spearman’s rank coefficient was used to study correlations between the predictors. Differences in weight change between dupilumab and other systemics were calculated with a mixed regression model. Interaction tests were performed.

When comparing measurements of tryptophan/kynurenine metabolites at baseline with those at follow-up, a non-parametric test was used (Wilcoxon’s signed-rank test). Correlations between weight change and change in metabolite levels were assessed using Spearman’s correlation test. No adjustment for multiple testing was performed.

All statistical analyses were performed in STATA/SE 17.0 statistical software (StataCorp, College Station, TX, USA).

## RESULTS

### Patients and baseline characteristics

We included 157 patients, who had their weight measured at the start of a relevant treatment period and at least once more during the same treatment period. The majority of patients (*n* = 126, 72%) were treated with dupilumab only, 39 (18%) were treated with another systemic medication, and 8 (10%) received both dupilumab and another systemic medication. Of the patients treated with other systemic treatments, 23 (59%) were treated with methotrexate, 11 (28%) with JAK inhibitors, and 5 (13%) with cyclosporine (**Table I**).

**Table I:** Baseline characteristics of patients with atopic dermatitis on systemic treatment

Factor	Dupilumab *n* = 126	Systemics *n* = 29	All cases *n* = 157
Age at start, years, median (IQR)	45.0 (30.8, 57.7)	31.1 (26.3, 47.1)	42.1 (29.9, 56.7)
Sex			
Male	60 (47.6%)	17 (44%)	74 (47.1%)
Female	66 (52.4%)	22 (56%)	83 (52.9%)
Age at onset			
< 2 years	55 (43.7%)	20 (51%)	72 (45.9%)
2–6 years	26 (20.6%)	12 (31%)	36 (22.9%)
7–12 years	5 (4.0%)	3 (8%)	6 (3.8%)
13–19 years	5 (4.0%)	0 (0%)	5 (3.2%)
≥ 20 years	15 (11.9%)	2 (5%)	16 (10.2%)
Missing	20 (15.9%)	2 (5%)	22 (14.0%)
Asthma			
No	51 (40.5%)	21 (54%)	66 (42.0%)
Yes	56 (44.4%)	16 (41%)	71 (45.2%)
Missing	19 (15.1%)	2 (5%)	20 (12.7%)
Allergy			
No	22 (17.5%)	8 (21%)	29 (18.5%)
Yes	85 (67.5%)	29 (74%)	108 (68.8%)
Missing	19 (15.1%)	2 (5%)	20 (12.7%)
Education**			
≤ 9 years	9 (7.1%)	4 (10%)	12 (7.6%)
> 9–12 years	25 (19.8%)	11 (28%)	34 (21.7%)
> 12 years	71 (56.3%)	22 (56.4%)	88 (56.1%)
Missing	21 (16%)	2 (6%)	23 (14.6%)
Current smoker			
No	96 (76.2%)	30 (77%)	120 (76.4%)
Yes	20 (15.9%)	8 (21%)	26 (16.6%)
Missing	10 (7.9%)	1 (3%)	11 (7.0%)
Weight (kg), mean (SD)	78.1 (21.2)	76.1 (21.6)	78.0 (21.3)
Body mass index (kg/m²), mean	26.6 (6.8)	25.1 (5.5)	26.3 (6.5)
Eczema Area Severity Index, median (IQR)	12.4 (7.0–23.0) (*n* = 102)	10.1 (4.6–15.1) (*n* = 31)	12.1 (6.9–22.0) (*n* = 126)
Dermatology Life Quality Index, median (IQR)	14.0 (7.0–20.0) (*n* = 111)	14.5 (9.0–20.0) (*n* = 26)	14.0 (8.0–20.0) (*n* = 130)
Patient-oriented eczema measure, median (IQR)	22.0 (17.0–26.0) (*n* = 109)	17.5 (15.0–24.0) (*n* = 269)	21.5 (17.0–26.0) (*n* = 128)
Visual analogue scale/Numeric rating scale, median (IQR)	7.0(5.5–8.2) (*n* = 104)	7.0 (5.0–8.0) (*n* = 24)	7.0 (5.4–8.0) (*n* = 122)
MADRS-S, median (IQR)	12.5 (4.5–20.0) (*n* = 96)	12.0 (6.0–19.0) (*n* = 21)	13.0 (4.5–20.0) (*n* = 112)

*Eight patients were treated with both types of medication.

** Patients aged 18–25 years included.

MADRS-S: Montgomery-Åsberg Depression Rating Scale-Self report.

### Weight change during treatment with dupilumab

**Fig. 1:**
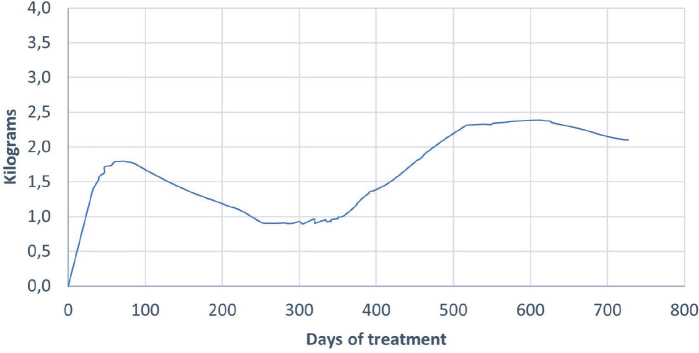
Weight change during treatment with dupilumab over 24 months.

On average, over 24 months, patients on dupilumab experienced a mean weight gain of 1.6 kg (*p* = 0.007, 95% confidence interval [CI] 0.4–2.7). Men gained 2.9 kg (*p* = 0.008, 95% CI 0.7–4.3) and women gained 0.7 kg (*p* = 0.318, 95% CI –0.7–2.0). Mean weight change at 6, 12, 18, and 24 months for patients treated with dupilumab was 2.2 kg (*n* = 33), 0.7 kg (*n* = 58), 2.8 kg (*n* = 36), and 2.3 kg (*n* = 11), respectively (**Fig. 1**).

### Predictors of weight change with dupilumab

In the bivariate analysis, only 1 of the 10 predictors, asthma, was statistically significant. It was inversely associated with weight gain during treatment with dupilumab (–2.8 kg, *p* = 0.013, 95% CI –4.94 to –0.60)). When stratified for asthma, the bivariate analysis for sex still showed non-significant results. In a fully adjusted model including all variables, asthma was the only significant predictor (–3.5 kg, *p* = 0.023, 95% CI –6.57 to –0.50). Male sex and BMI tended to be significantly associated with weight change, although the change in weight was small (**Table II**).

**Table II:** Predictors of weight change during dupilumab treatment

Predictors	Unadjusted bivariate analysis (*p*-value)	Fully adjusted analysis	Adjusted analysis
Male sex	1.80 kg (*p* = 0.11)	2.55 kg (*p* = 0.07)	1.65 kg (*p* = 0.10)
BMI	–0.03 kg (*p* = 0.06)	–0.17 kg (*p* = 0.23)	–0.22 kg (*p* = 0.075)
Asthma	–2.80 kg **(*p* = 0.01)**	–3.54 kg **(*p* = 0.02)**	–2.41 kg **(*p* = 0.02)**
Age	–0.06 kg (*p* = 0.15)	–0.04 kg (*p* = 0.24)	–0.04 kg (*p* = 0.16)
MADRS-S	–0.03 kg (*p* = 0.98)	–0.07 kg (*p* = 0.33)	–
EASI	–0.05 kg (*p* = 0.47)	–0.07 kg (*p* = 0.21)	–
POEM	0.04 kg (*p* = 0.55)	0.19 kg (*p* = 0.09)	–
DLQI	0.02 kg (*p* = 0.67)	–0.03 kg (*p* = 0.80)	–
Allergy*	1.2 kg (*p* = 0.59)	0.55 kg (*p* = 0.67)	–
VAS/NRS	–0.00 kg (*p* = 0.94)	0.00 kg (*p* = 0.22)	–

* Reported rhinitis and/or conjunctivitis.

BMI: body mass index; DLQI: Dermatology Life Quality Index; EASI: Eczema Area Severity Index; MADRS-S: Montgomery-Åsberg Depression Rating Scale-Self report; NRS: numeric rating scale; POEM: patient-oriented eczema measure; VAS: visual analogue scale. Significant values in bold text.

### Patients treated with dupilumab compared with patients treated with other systemic medications

Over 24 months, the crude mean weight change in patients treated with dupilumab was 2.0 kg higher (*p* = 0.030, 95% CI 0.2–4.0), than that in patients on other systemic medications. Stratified by the different systemic treatments, patients on JAK inhibitors (*n* = 11) had a mean weight change of 1.6 kg (*p* = 0.2), patients on methotrexate (*n* = 23) one of 0.3 kg (*p* = 0.7), and patients on cyclosporine (*n* = 5) one of –1.6 kg (*p* = 0.7).

In the multivariable analysis, controlling for confounders (age at start, sex, BMI at start, and asthma), dupilumab was associated with a mean weight gain of 3.3 kg (*p* = 0.005, 95% CI 1.0–5.6), compared with other systemic treatments. We found that asthma had an inverse association with weight gain. Therefore, we performed a sensitivity analysis excluding patients who had reported asthma. Patients without reported asthma who were treated with dupilumab gained 4.8 kg more in weight (*p* = 0.005, 95% CI 1.5–8.2), than patients on other systemics. There was no interaction between dupilumab treatment and asthma when tested.

### Correlation between weight change and outcome measures for patients treated with dupilumab

There was no statistically significant correlation between weight change and change in VAS/NRS, EASI, POEM, DLQI, MADRS-S, the sleep component of POEM, or the hunger component of MADRS-S (**Table III**).

**Table III:** Spearman rank correlation coefficients between change in weight and relevant variables during treatment with dupilumab

Variable	Spearman rank correlation (*p*-value)
Disturbed night sleep due to itch*	–0.066 (*p* = 0.9)
Hunger/appetite**	–0.09 (*p* = 0.30)
MADRS-S	0.09 (*p* = 0.80)
POEM	0.02 (*p* = 0.80)
DLQI	–0.05 (*p* = 0.54)
VAS/NRS	0.10 (*p* = 0.2)
EASI	0.10 (*p* = 0.74)

* Reported in POEM.

** Reported in MADRS-S.

DLQI: Dermatology Life Quality Index; EASI: Eczema Area Severity Index; MADRS-S: Montgomery-Åsberg Depression Rating Scale-Self report; NRS: numeric rating dcale; POEM: patient-oriented eczema measure; VAS: visual analogue scale.

### Tryptophan/kynurenine metabolites in relation to weight change during dupilumab treatment

Most AD patients (*n* = 13) treated with dupilumab who had provided blood before treatment and during follow-up gained weight during the follow-up (median 5.5 kg, interquartile range [IQR] 2.3–11.0, mean 8.1 kg, *p* = 0.002). The weight gain was lower at the final follow-up, as several patients who had gained weight subsequently lost weight, but still significant (median 1.3 kg, IQR 0.0–5.4, mean 3.6 kg, *p* = 0.045). For the 33 different tryptophan/kynurenine metabolites, the median concentration did not change significantly from baseline to follow-up (Table SI). There were no significant correlations between changes in tryptophan/kynurenine metabolites and weight change (Appendices S1 and S2).

## DISCUSSION

Individuals with AD are not commonly identified as living with overweight or obesity (22), yet our results showed that the mean weight gain with dupilumab was 1.6 kg during the first 24 months of treatment, with males showing a tendency to gain more weight than females. In addition, dupilumab patients gained more weight (3.3 kg) than patients treated with other systemic medications. Given that the weight gain was not correlated with eczema improvement, it could be a direct effect of the dupilumab treatment. Asthma was associated with weight loss and the only significant predictor of weight change. Male sex showed a tendency towards correlation with weight gain, but no significant associations or correlations with other predictors were observed.

The mean weight gain of 1.6 kg is larger than expected in the Swedish population over 2 years. A Swedish cohort study shows that BMI has increased over a 24-year period in Sweden, with mean BMI rising from 24.1 to 25.5 for men and from 23.1 to 24.3 for women. Calculating based on average heights for Swedish men and women, this equates to a mean weight increase of approximately 3.5 kg for men and 3.3 kg for women over the study period, or about 0.3 kg every 2 years. This suggests that the expected natural weight gain over time in the general population is relatively modest and smaller than found in our study (23).

Dupilumab is used by allergologists to treat asthma (24) and dermatologists often choose this medication for patients with atopic comorbidities (7). In our study, 45% of the patients in the dupilumab group reported having had asthma at some point in life. It is possible that the weight loss associated with asthma can be explained by improvement of asthmatic symptoms and a reduced need for inhaled corticosteroids (25).

The mechanisms of the weight gain in this context are largely unknown. Earlier studies have speculated that reduced itching and decreased scratching during sleep may lead to lower energy expenditure (26). In our study, we did not find any associations between weight change and reported sleep loss due to itching or reported changes in appetite. In addition, we did not see any correlation between weight change and assessed improvement of eczema.

Transcriptome analyses of skin samples in patients treated with dupilumab suggest decreased IL-13 levels during treatment (27); this could be a possible mechanism of weight change. It has been reported that IL-4 and IL-13 depletion induces a reduction in brown adipose tissue, which would in turn reduce systemic energy expenditure. This is proposed to be due to interference with adipose tissue metabolism, as preventing the action of IL-4/IL-13 on adipose tissue macrophages would reduce their M2 activation. Enrichment in adipose tissue-resident M2 macrophages correlates with higher insulin sensitivity and energy expenditure (28).

Another explanation could be that reduced activation of IL-4I1 decreases the levels of indole compounds and KA (13). KA has been shown to increase adipose tissue energy expenditure and reduce circulating triglyceride levels (14). Furthermore, IL4i1 downstream of the IL-4/IL-4 receptor can generate KA outside the conventional kynurenine pathway (14). However, our analysis found no significant links between changes in tryptophan/kynurenine metabolites and weight change. This suggests that, in this context, the conventional kynurenine pathway adequately maintains physiological KA levels.

In this real-life cohort study, all data were prospectively collected at dermatology clinics in Sweden. The outcome measures, based on HOME recommendations, were uniformly designed throughout the clinics. Patients registered their PROMs on tablets or smartphones and could answer in privacy, reducing interviewer bias.

### Limitations

The present study has several limitations, including potential sources of information bias, particularly concerning the reporting of age at onset, asthma diagnosis, and prior allergies. There is also a risk of confounding by indication (e.g., dupilumab is more often chosen for patients with asthma comorbidity). Additionally, the lack of mandatory weight measurements contributed to a smaller study sample than expected. Some of our predictors may be of importance for weight gain but failed to reach significance in our study due to sample size. Due to differences in follow-up routines across dermatological clinics, patient data may have been obtained at different time intervals and frequency during the study period. This may have contributed to the biphasic distribution in weight, shown in Fig. 1, as the included participants differed between the times of measurement.

### Conclusion

AD patients treated with dupilumab gained weight significantly over the course of 24 months. Patients without reported asthma had greater weight gain. Concentrations of tryptophan/kynurenine metabolites were not associated with weight gain and further studies are needed to understand the mechanism of the weight gain during dupilumab treatment.

## Supplementary Material

Risk Factors Associated with Weight Gain during Treatment with Dupilumab among Patients with Moderate to Severe Atopic Dermatitis

Risk Factors Associated with Weight Gain during Treatment with Dupilumab among Patients with Moderate to Severe Atopic Dermatitis

Risk Factors Associated with Weight Gain during Treatment with Dupilumab among Patients with Moderate to Severe Atopic Dermatitis
