# Supplementary material for: Risk Factors Associated with Weight Gain during Treatment with Dupilumab among Patients with Moderate to Severe Atopic Dermatitis
Source: Acta Derm Venereol. 2024 Nov 15;104:40796. doi: 10.2340/actadv.v104.40796 (PMC11586677; doi:10.2340/actadv.v104.40796)
Supplement: Risk Factors Associated with Weight Gain during Treatment with Dupilumab among Patients with Moderate to Severe Atopic Dermatitis [file ActaDV-104-40796-s1.pdf]

Supplementary material has been published as submitted. It has not been copyedited, or typeset by Acta Dermato-Venereologica

APPENDIX S1

S1 Correlation between change in metabolites and change in weight

Spearman's rank correlation coefficient calculated for each metabolite (difference between baseline and follow-up) and visualized in scatterplot with weight change. No significant associations between change in tryptophan/kynurenine metabolites and weight change.

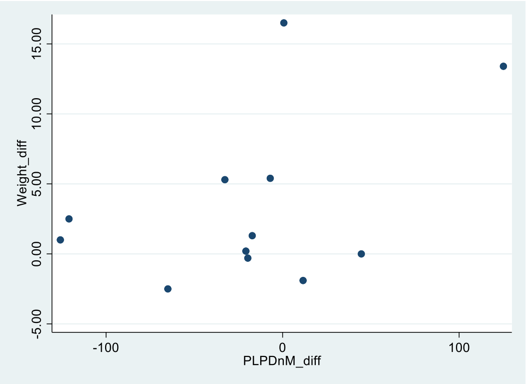

Spearman's rho = 0.2098, p = 0.5128.

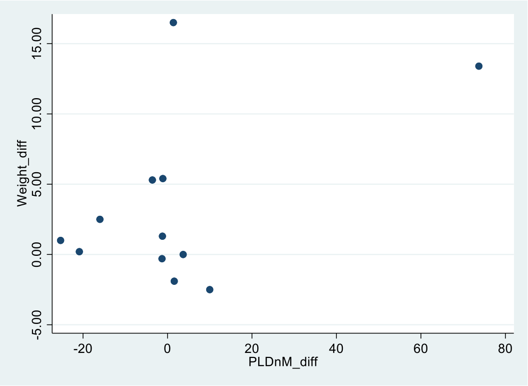

Spearman's rho = -0.0490, p = 0.8799.

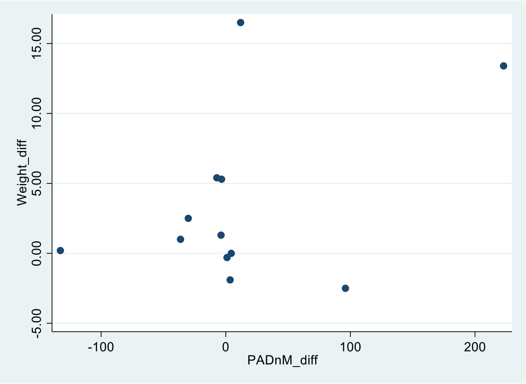

Spearman's rho = -0.0070, p = 0.9828.

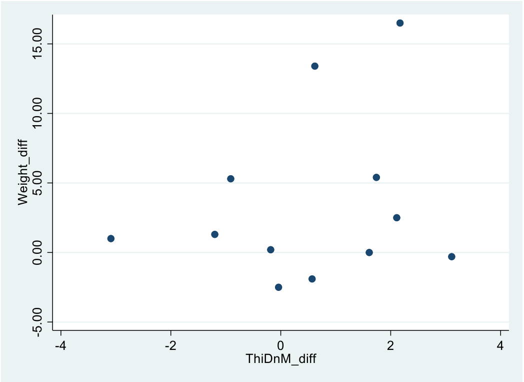

Spearman's rho = 0.1678, p = 0.6021.

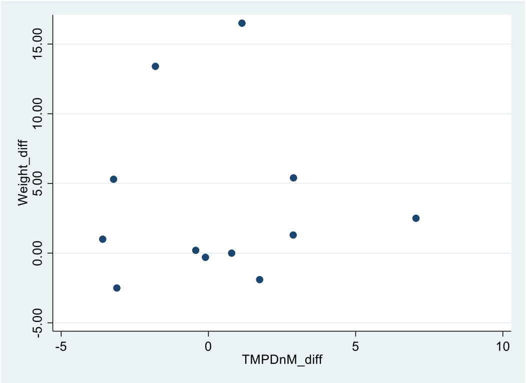

Spearman's rho = 0.1748, p = 0.5868.

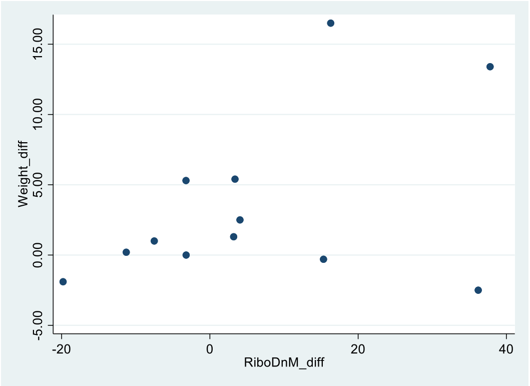

Spearman's rho = 0.3147, p = 0.3191.

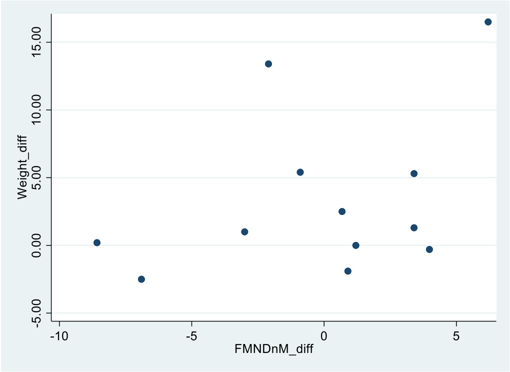

Spearman's rho = 0.2452, p = 0.4424.

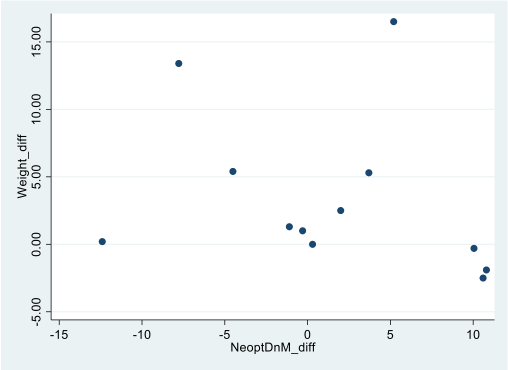

Spearman's rho = -0.4685, p = 0.1245.

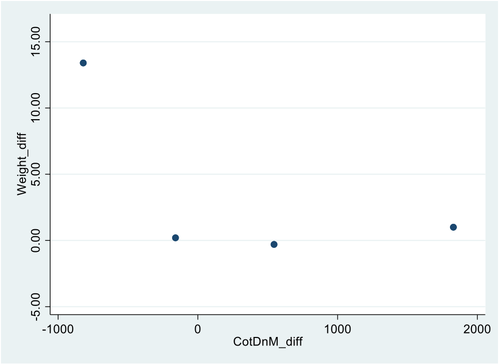

Spearman's rho = -0.4000, p = 0.6000.

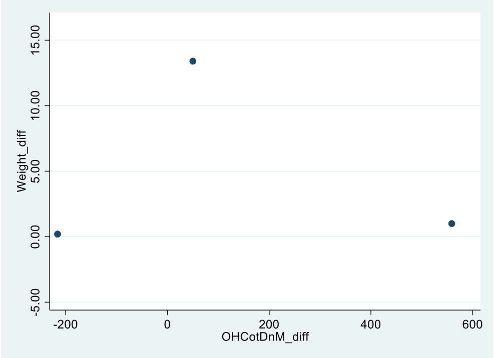

Spearman's rho = 0.5000, p = 0.6667.

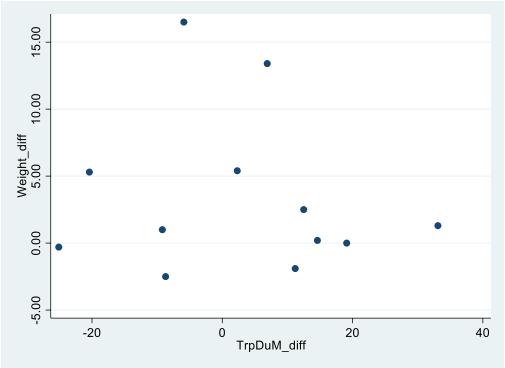

Spearman's rho = -0.0070, p = 0.9828.

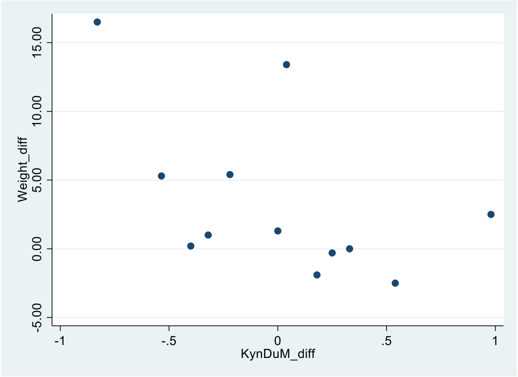

Spearman's rho = -0.5524, p = 0.0625.

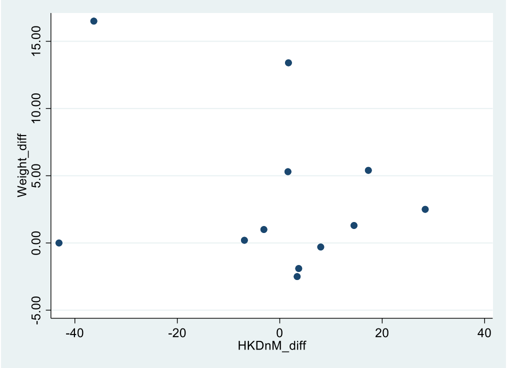

Spearman's rho = -0.0210, p = 0.9484.

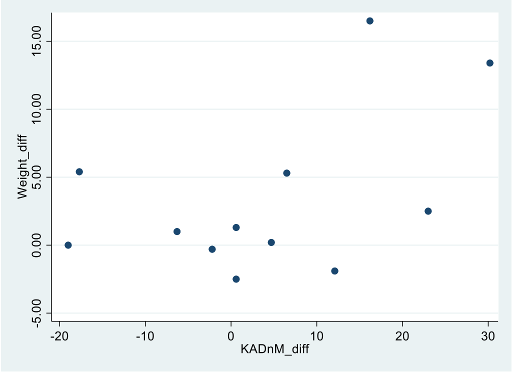

Spearman's rho = 0.3923, p = 0.2072.

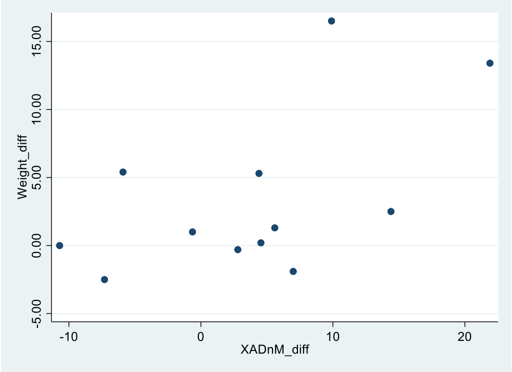

Spearman's rho = 0.4965, p = 0.1006.

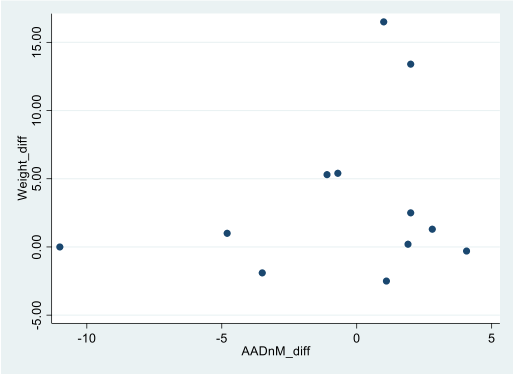

Spearman's rho = 0.0946, p = 0.7700.

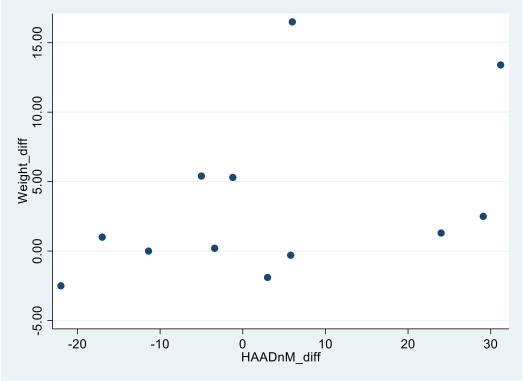

Spearman's rho = 0.5105, p = 0.0899.

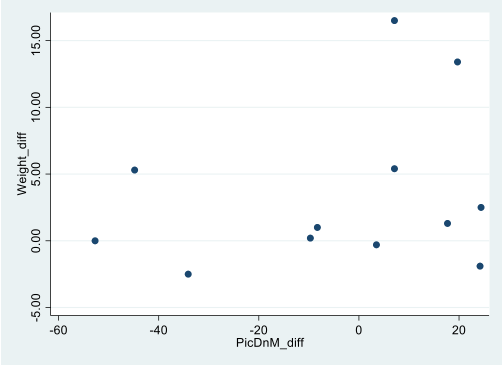

Spearman's rho = 0.2942, p = 0.3533.

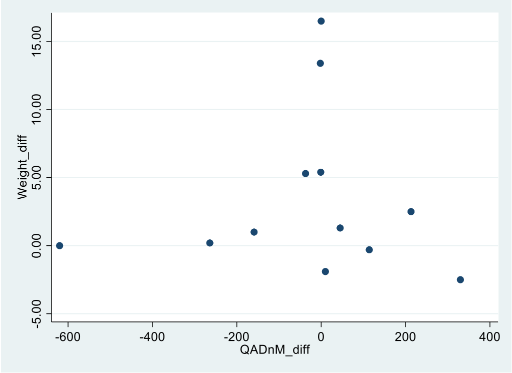

Spearman's rho = -0.2168, p = 0.4986.

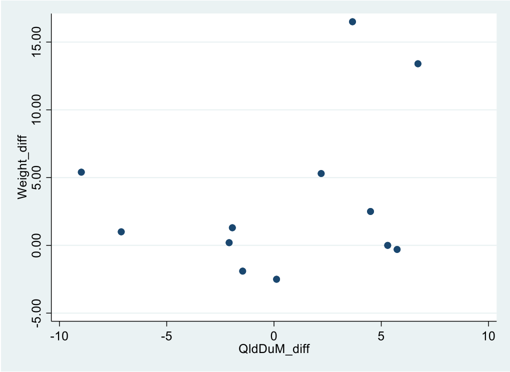

Spearman's rho = 0.0699, p = 0.8290.

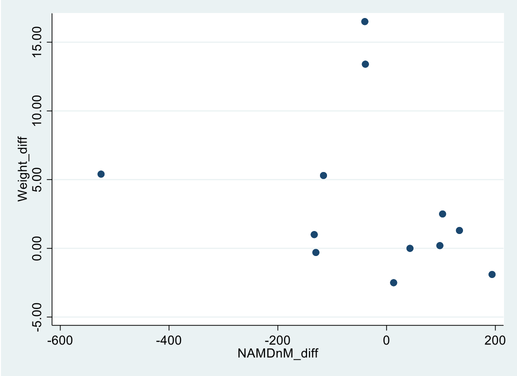

Spearman's rho = -0.3427, p = 0.2756.

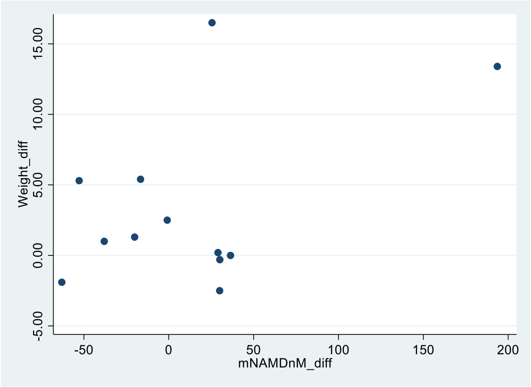

Spearman's rho = -0.0280, p = 0.9312.

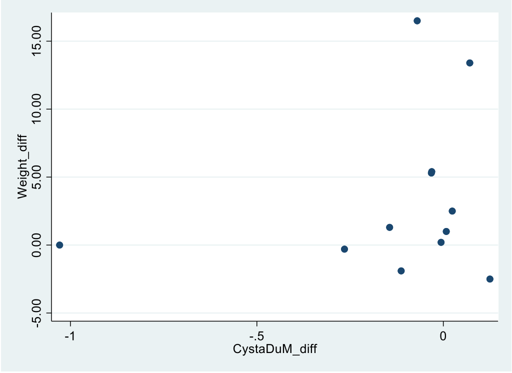

Spearman's rho = 0.1608, p = 0.6175.

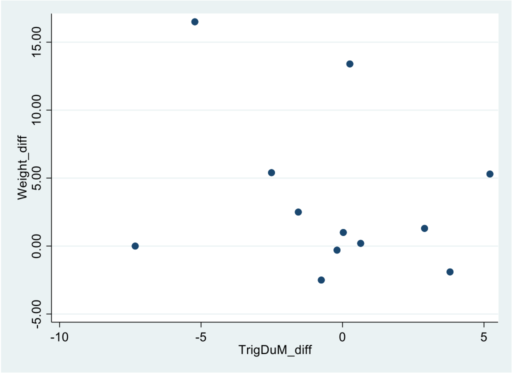

Spearman's rho = -0.1329, p = 0.6806.

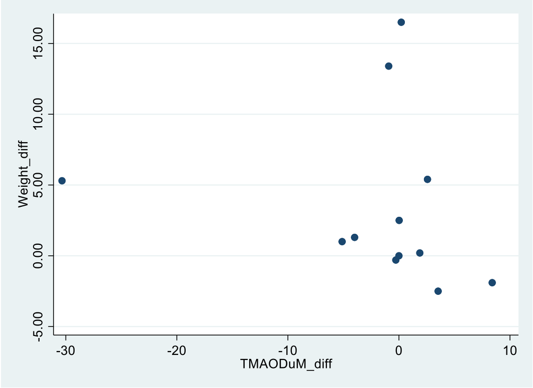

Spearman's rho = -0.3497, p = 0.2652.

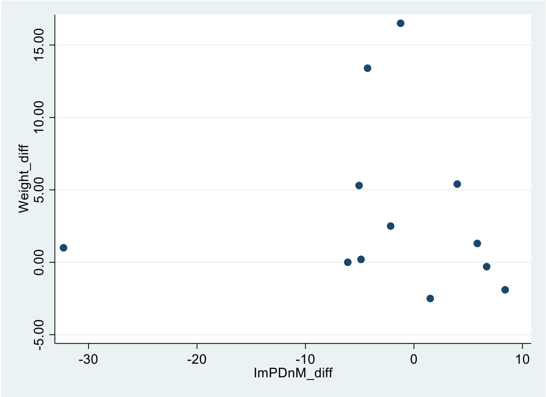

Spearman's rho = -0.2378, p = 0.4568.

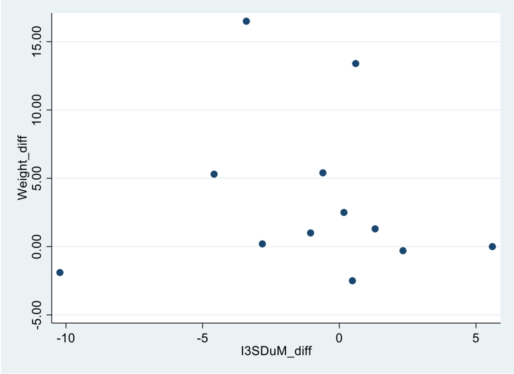

Spearman's rho = -0.1888, p = 0.5567.

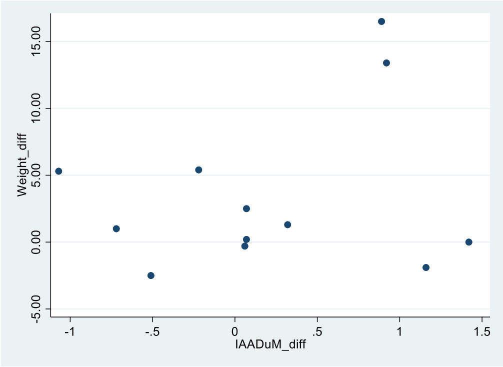

Spearman's rho = 0.0035, p = 0.9914.

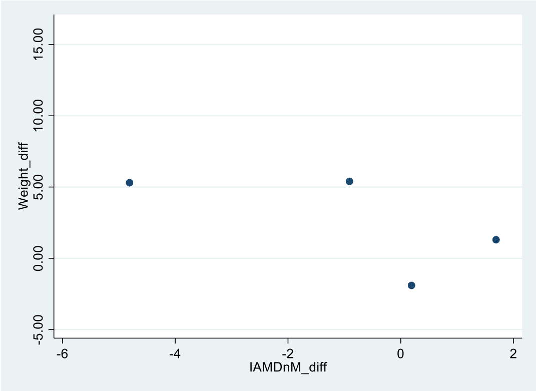

Spearman's rho = -0.6000 (n = 4), p = 0.4000.

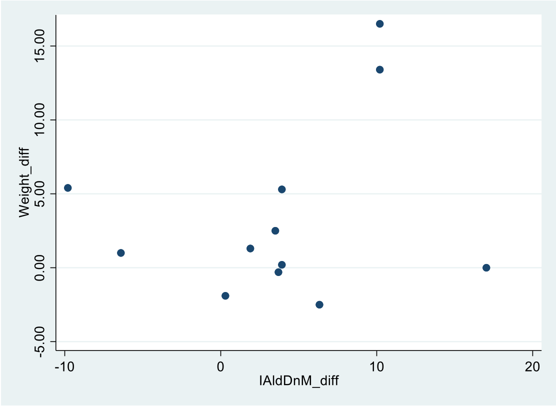

Spearman's rho = 0.0737, p = 0.8200.

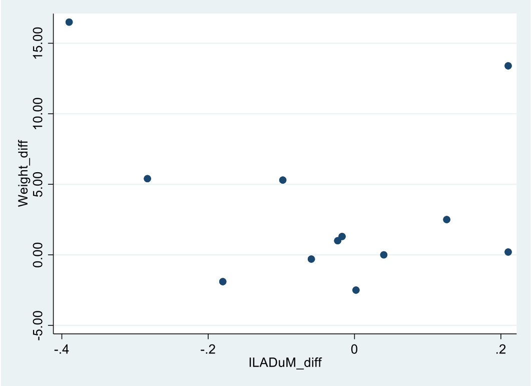

Spearman's rho = -0.1751, p = 0.5862.

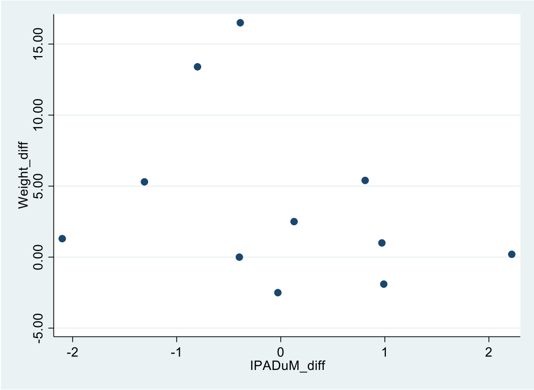

Spearman's rho = -0.3727, p = 0.2589.

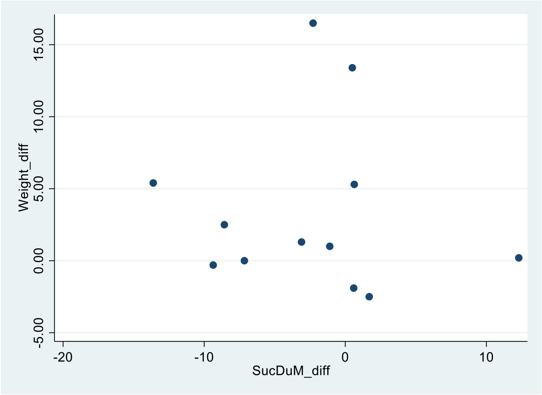

Spearman's rho = -0.2448, p = 0.4433.

APPENDIX S2

S2 Correlations with tryptophan/kynurenine metabolites

Spearman's rank correlation coefficient calculated for each metabolite (n = 33) (difference in concentration between baseline and follow-up and/or metabolite concentration at baseline) and analyzed in relation to different outcomes. Adjustment for multiple testing was not performed.

Correlation between max weight change during follow-up and change in tryptophan/kynurenine metabolites

There was one significant correlation between maximum weight change during follow-up and change in the metabolite cystathionine (Cysta) (n = 12, rho = 0.5944, p = 0.0415).

Correlation between weight at baseline and tryptophan/kynurenine metabolites at baseline

There was one strong and significant negative correlation between weight at baseline and the metabolite indole-3-acetamide (IAM) at baseline (n = 7, rho = -0.8929, p = 0.0068).

Correlation between weight at baseline and change in tryptophan/kynurenine metabolites

No significant associations.

Correlation between baseline EASI and tryptophan/kynurenine metabolites at baseline

There were three significant negative correlations between EASI and metabolites at baseline: picolinic acid (Pic) (n = 12, rho = -0.6620, p = 0.0190), quinaldic acid (Qld) (n = 12, rho = -0.6351, p = 0.0265), trigonelline (Trig) (n = 12, rho = -0.5884, p = 0.0441).

Correlation between change in EASI and change in tryptophan/kynurenine metabolites

There was one significant correlation between change in EASI and change in the metabolite N1-methylnicotinamide (mNAM) (n = 12, rho = 0.6434, p = 0.0240).

Correlation between weight and AD outcomes

No significant correlation between eczema outcome measurement and weight change.

There were no associations between change in weight and changes in EASI, POEM, pruritus-NRS, or MADRS-S.

AD severity (EASI, POEM, pruritus-NRS, MADRS-S) at baseline was not associated with weight change.
