# Supplementary figures and images for: Risk Factors Associated with Weight Gain during Treatment with Dupilumab among Patients with Moderate to Severe Atopic Dermatitis
Source: Acta Derm Venereol. 2024 Nov 15;104:40796. doi: 10.2340/actadv.v104.40796 (PMC11586677; doi:10.2340/actadv.v104.40796)

Fig. S1. Flowchart of cohort selection.

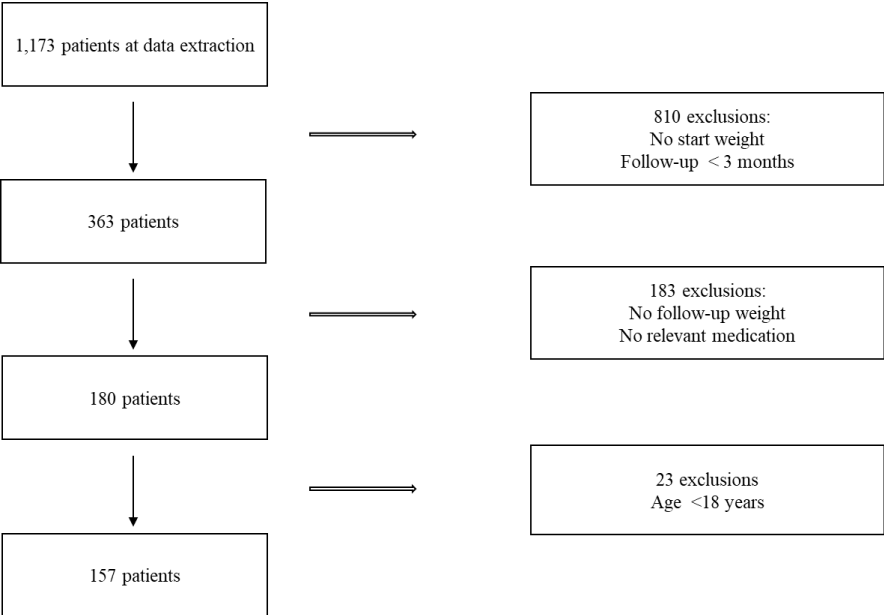

Supplement: Risk Factors Associated with Weight Gain during Treatment with Dupilumab among Patients with Moderate to Severe Atopic Dermatitis [file ActaDV-104-40796-s2.pdf]
